# Mechanical ventilation in a case of anticoagulant rodenticide toxicity: a case report

**DOI:** 10.3389/fvets.2026.1762708

**Published:** 2026-02-25

**Authors:** Diana Carter, Carl J. Southern

**Affiliations:** University of Florida College of Veterinary Medicine, Gainesville, FL, United States

**Keywords:** anticoagulant rodenticide, case report, critical care, emergency, mechanical ventilation, novel therapy, veterinarian medicine

## Abstract

A 2.5-year-old male neutered Golden Retriever was taken to an academic teaching hospital in respiratory distress. The patient was intubated, and diagnostics were performed, which found delayed clotting times and evidence of hemorrhage in both his trachea and lungs. The owner mentioned the possibility of anticoagulant rodenticide exposure. Due to upper and lower airway impairment, the patient was mechanically ventilated for more than 24 h while standard treatment was initiated. The patient was able to breathe on his own after 24 h and was discharged home 4 days later. This report documents a dog that required mechanical ventilation due to suspected intramural tracheal membranous hemorrhage and atypical presentation of upper and lower airway hemorrhage caused by vitamin K antagonist rodenticide.

## Introduction

Anticoagulant rodenticides have been one of the most common presenting problems in veterinary emergency rooms over the last 10 years (1–4). Two generations of anticoagulant rodenticides differ in their potency and duration of action. Rodent resistance to first-generation anticoagulant rodenticides (warfarin, diphacinone, coumatetralyl, dicoumarol, chlorophacinone, and coumafuryl) resulted in the development of more potent second-generation drugs (brodifacoum, bromadiolone, difenacoum, flocoumafen, and difethialone). Anticoagulant rodenticides inhibit the vitamin K epoxide reductase enzyme, thereby preventing the recycling of vitamin K into its active form, which is required as a cofactor for the synthesis of coagulation factors II, VII, IX, and X (5). The most common adverse event associated with anticoagulant rodenticide toxicity is hemorrhage, which may be severe. Bleeding can occur in the gastrointestinal and/or urinary tracts, but it can also occur intracranially or in the pulmonary parenchyma. All of these conditions may rapidly become life-threatening. Specific clinical signs include pulmonary hemorrhage and cavitary bleeding, while non-specific symptoms include weakness, respiratory distress, and collapse. The traditional treatment for anticoagulant rodenticide toxicity consists of administering vitamin K alongside fresh frozen plasma (FFP), which replaces the vitamin K-dependent coagulation factors depleted by the antagonist mechanism. With timely therapy, the prognosis can be excellent (6).

This case report describes the treatment of a dog that required mechanical ventilation due to suspected intramural tracheal membranous hemorrhage and atypical presentation of upper and lower airway hemorrhage caused by a vitamin K antagonist rodenticide.

## Case description

A 2.5-year-old male neutered Golden Retriever presented to a tertiary referral academic institution’s emergency room in respiratory distress. Two nights prior to presentation, the patient developed coughing that progressed to hemoptysis. The following day, the patient coughed/hacked all night and was visited by his primary care veterinarian in the morning. At this time, he was in respiratory distress, as described by tachypnea and significant abdominal effort on inspiration. There was no previous medical or surgical history. He was referred to a tertiary referral academic institution for additional diagnostics, supportive care, and treatment. On physical exam, he remained in respiratory distress (110 breaths per minute), tachycardic (166 beats per minute), and was ultimately sedated and intubated shortly after his exam due to concern about respiratory fatigue. Once intubated, the owners told clinicians there was a risk of exposure to vitamin K antagonist rodenticide (brodifacoum) on the property, which had been previously placed underneath the house 1 year prior.

**Figure 1:**
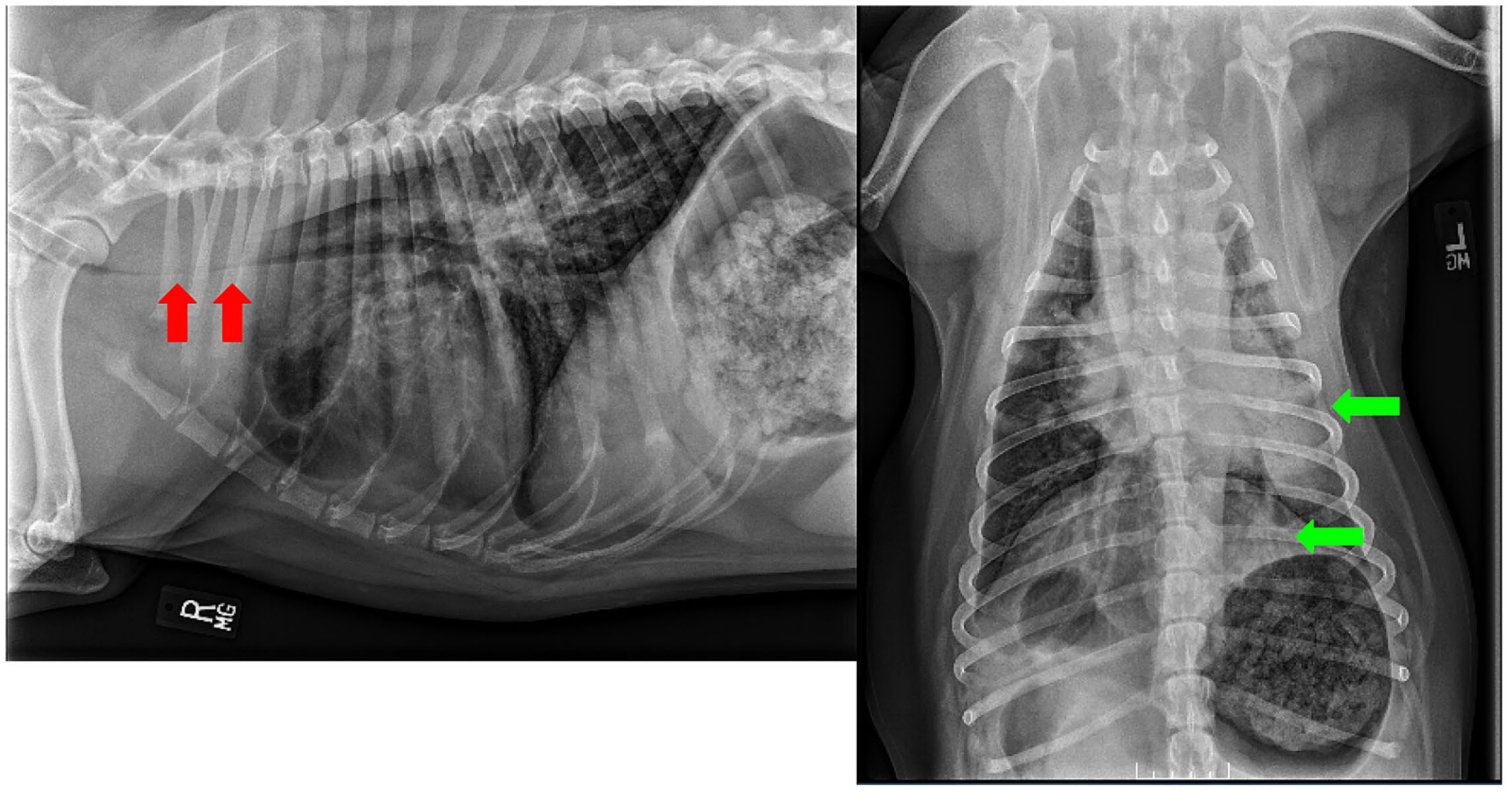
Thoracic radiographs from the patient on intake post-intubation.

Initially, several differential diagnoses were considered as potential causes of his respiratory distress. His high inspiratory effort raised the possibility of laryngeal paralysis, which is typically found in older dogs. Given the patient’s degree of respiratory effort and tachypnea, infectious disease of the pulmonary parenchymal tissues (bacterial, viral, fungal, or aspiration) was evaluated. Due to the patient’s severe respiratory fatigue and cyanosis, the patient was intubated. Bloodwork and thoracic radiographs were performed after the patient was successfully intubated. During intubation, localized hemorrhages were seen around the laryngeal muscles and throughout the epiglottis. Chest radiographs (Figure 1) were performed at the time of presentation and revealed a significantly decreased tracheal diameter (red arrows) and increased soft tissue opacity, suggesting a mass-like effect due to tissue or fluid, such as blood. Additionally, there was a multifocal, predominantly left-sided unstructured interstitial alveolar pulmonary pattern (green arrows). This was suggestive of pulmonary edema (secondary to upper airway obstruction), atypical infectious disease-causing pneumonia, or coagulopathy, which resulted in hemorrhage into both the lung parenchyma and the esophagus (Figure 1). During intubation, the visualization of hemorrhage around the larynx confirmed what was suspected, based on the radiographs. Comprehensive bloodwork was simultaneously pursued. A complete blood count (CBC) revealed mild neutrophilia (9.07; 2.6–8.0 k/L), mild lymphopenia (0.65; 1.0–4.0 k/L), and thrombocytopenia (94; 130–328 K/μL) with a few large platelets clumped along the blood smear. A chemistry panel found mildly increased creatine kinase (441: 49–244 U/L), hyperglycemia (153; 78–124 g/dL), hypokalemia (3.1; 3.8–5.0 mEq/L), and mild hypochloremia (107.6; 107.8–117.1 mEq/L). Importantly, a coagulation panel was performed, which revealed severely elevated prothrombin time (PT) (>70 s; 7.06–9.00) and mildly elevated partial thromboplastin time (aPTT) (24 s; 10.12–14.21). The patient’s blood type was found to be DEA 1, and a comprehensive rodenticide panel was sent to an outside laboratory, with results expected within 8 days, testing for exposure to brodifacoum, bromadiolone, chlorphacinone, dicoumarol, difenacoum, difethialone, diphacinone, and warfarin.

Because of the coagulation panel results, his age, and possible exposure to anticoagulant rodenticide, the patient was tentatively diagnosed with anticoagulant toxicity and was promptly treated with fresh frozen plasma IV (500 mL/15 mL/kg as a bolus then 100 mL/h for 4 h), vitamin K subcutaneous injection (5 mg/kg), tranexamic acid IV (10 mg/kg) every 8 h, and a dose of antibiotics (ampicillin/sulbactam 30 mg/kg IV) (given preemptively prior to bloodwork results). The patient remained intubated and was transferred to the ICU for advanced respiratory support using positive-pressure mechanical ventilation. The patient was started on synchronized intermittent mechanical ventilation (SIMV) with pressure support, and he required positive pressure mechanical ventilation for just over 24 h before being weaned off the ventilator and successfully maintained on room air. The initial settings included a respiratory rate of 15 breaths per minute, a PEEP of 3 cmH_2_O, a tidal volume of 8 mL/kg (gradually increased to 10 mL/kg), 100% FiO_2_, and a peak pressure of 10 cmH_2_O. During the first 8 h of ventilation, the respiratory rate was increased to 25 breaths per minute, FiO_2_ was gradually weaned to 40%, and pressure support was reduced to allow for more spontaneous efforts with less support. He was monitored q1 for end tidal carbon dioxide, SpO_2_, FiO_2_, blood pressure, temperature, respiratory rate, and heart rate while on ventilation. Furthermore, his mouth was suctioned every 4 h and evaluated for hemorrhage. Following intubation and initial airway suctioning, subsequent suction attempts were largely ineffective, yielding minimal to no visible hemorrhage. Over 24 h later, he was successfully weaned off the ventilator and continued his recovery in room air until discharge.

After the patient was extubated and successfully weaned from the ventilator, he was able to tolerate oral medications including oral antibiotics (amoxicillin/clavulanic acid at 17 mg/kg PO q12 h), vitamin K (2.5 mg/kg PO q12 h) for a total of 28 days and other supportive gastrointestinal medications (cisapride 0.45 mg/kg PO q8–12 h; maropitant 1.8 mg/kg PO q24 h) to help reduce nausea and maintain appetite. His coagulation panel (PT/aPTT, Table 1) was repeated daily until discharge, and the results showed that the values were improving, which correlated with improving clinical signs. The patient was then discharged from the hospital 4 days later and, according to owner communication, was recovering uneventfully. The results of the rodenticide panel confirmed toxicity and detected levels of brodifacoum in the submitted sample.

**Table 1:** Daily coagulation panel results.

Coagulation parameter (reference range)	Day 1 (1/26)	Day 2 (1/27)	Day 3 (1/28)
PT (7.0–9.0) s	>70	9.4	8.4
aPTT (10.12–14.21) s	24.8	15.2	13.8

## Discussion

This case report describes the novel usage of mechanical ventilation as a supportive measure for anticoagulant rodenticide toxicity due to upper respiratory obstruction from hemorrhage suggestive of pulmonary parenchymal disease, most likely pulmonary hemorrhage. Anticoagulant rodenticide toxicity patients may exhibit symptoms such as lethargy, pale mucous membranes, inappetence, dyspnea, and coughing with petechiae, ecchymosis, epistaxis, or hematemesis, followed by hemorrhage of various tissues, including the pericardium (6, 7). However, some patients who are bleeding into the thoracic cavity may present with respiratory distress or display acute dyspnea. Hemorrhaging can occur in both the pleural space and the pulmonary parenchyma, reducing the lungs’ capacity for ventilation. In a recent retrospective paper, pulmonary hemorrhage causing a hemothorax was the most reported site of bleeding (8). Simultaneously, imaging revealed upper airway obstruction due to suspected dorsal tracheal membrane hemorrhage, which is uncommon. One case series reported upper airway obstructions in five dogs who were misdiagnosed due to the atypical presentation, but none required mechanical ventilation (9). Collectively, evidence of upper and lower airway disease leads to patients demonstrating signs of cyanosis and respiratory fatigue requiring intubation and can ultimately lead to mechanical ventilation.

The three main indications for mechanical ventilation are severe hypoxemia despite oxygen supplementation, severe hypoventilation despite therapy, and excessive respiratory effort with impending respiratory fatigue or failure (10). While arterial blood gases can be an indicator of oxygenation status, there is no objective measurement of respiratory fatigue, and it is reliant on the clinician’s judgement. The clinician elected to proceed directly with mechanical ventilation rather than trial high-flow nasal oxygen, which would also have been an appropriate subsequent treatment option. Positive pressure mechanical ventilation improves oxygenation by recruiting alveoli for gas exchange, but it can potentially be detrimental to the lungs and cause complications such as ventilator-associated pneumonia, pneumothorax, acute lung injury, and acute respiratory distress syndrome (ARDS), which can occur and affect prognosis. The primary disease process is typically the major influencer of the prognosis of a patient successfully weaning off the ventilator and surviving hospitalization to discharge (11).

Mechanical ventilation of a patient can be laborious for medical staff and is often cost-restrictive for owners. In this case report, the cause of the patient’s respiratory disease was theoretically temporary, and mechanical ventilation was utilized until the patient could be successfully weaned off the ventilator. In this case report, repeat imaging 48 h later revealed improved oxygenation saturation as well as the resolution of infiltrates in his thoracic and tracheal cavities.

Mechanical ventilation allowed clinicians to both secure the airway and allow for direct suction of hemorrhage from the upper airways. It also provided supplemental oxygen and reduced the work of breathing for this patient while traditional therapy (plasma and vitamin K) had time to become effective. Lower tidal volumes were utilized initially while the patient was ventilated to minimize volutrauma and hemorrhage, but they were gradually increased throughout the ventilatory period until extubation.

Anticoagulant rodenticides result in vitamin K antagonism, which causes a depletion of vitamin K-dependent coagulation factors II, VII, IX, and X (7). Onset of clinical signs occurs once coagulation factors have been depleted (typically within 3–5 days after ingestion). In cases of life-threatening hemorrhage, active clotting factors are restored through the transfusion of blood products (whole blood, fresh-frozen plasma, frozen plasma, or cryo-poor plasma). Frozen plasma contains specific vitamin K-dependent clotting factors (II, VII, IX, and X), which are decreased during toxicity (12). Vitamin K or phytonadione is a fat-soluble cofactor that uniquely activates coagulation factors (II, VII, IX, and X), as well as proteins C&S. Endogenous clotting factors produced by the body may be activated within 3–6 h with prothrombin times normalizing within 12 h after administration of vitamin K. Treatment in various formulations (intravenous, orally, and subcutaneously) has been studied, resulting in oral and IV dosing providing effective reduction in anticoagulant levels in humans within 24 h, whereas subcutaneous administration was less effective (13). It is currently unknown how quickly subcutaneous dosing of vitamin K can reverse coagulopathies due to rodenticide in dogs (14). A recent canine investigation found that IV administration of vitamin K may treat toxicosis and activate clotting factors within 1 h (15). However, it has already been reported that intravenous formulations lead to anaphylactoid reactions due to their composition and solvents (16, 17). While newer studies have demonstrated safe administration with intravenous dosing with new formulations, they are not always readily available, so subcutaneous vitamin K remains a safe alternative until patients can tolerate oral administration of the medication with a fatty meal to maximize absorption (14). Typically, recovery from life-threatening hemorrhage by the administration of exogenous clotting factors and vitamin K may begin within the first 48 h and in this patient’s case, it was paramount to the patient being successfully weaned off the ventilator.

## Conclusion

Anticoagulant rodenticide toxicity can present atypically and lead to respiratory distress by causing obstruction of the upper and lower airways, resulting in respiratory failure.

In this case report, temporary mechanical ventilation was necessary until traditional treatment with procoagulants and other supportive care could be implemented. Despite the severe respiratory compromise, cases that have evidence of upper and lower respiratory compromise may have a good prognosis with urgent and effective treatment.

## Data Availability

The original contributions presented in the study are included in the article/supplementary material, further inquiries can be directed to the corresponding author.
